# Supplementary material for: Prevalence and risk factor for mistreatment in childbirth: In health facilities of Gondar city, Ethiopia
Source: PLoS One. 2022 May 5;17(5):e0268014. doi: 10.1371/journal.pone.0268014 (PMC9070956; doi:10.1371/journal.pone.0268014)
Supplement: S1 Table — (PDF) [file pone.0268014.s001.pdf]

Category and types of mistreatment	Experienced of mistreatment	
	No (%)	Yes (%)
**Physical abuse**		
Health provider(s) physically hit, slapped, pushed, pinched or otherwise beat you?	542(94.4%)	32(5.6%)
Verbally (insulting) abuse during labor or delivery?	511(89.00%)	63(11%)
Separate mother from baby without medical indication?	566(98.60%)	8(1.4%)
Support staffs insult me and my companion?	492(85.70%)	82(14.3%)
Receiving unnecessary uncomfortable/pain-relief treatment?	560(97.60%)	14(2.4%)
Denied from food or fluid in labor unless medically necessitated?	574(100%)	0(0%)
**Non confidential care**		
The providers use drapes or covering appropriate to protect mother's privacy?	415(72.30%)	159(27.7%)
Health providers discussed your private health information in a way that others could hear?	554 (96.50%)	20(3.5%)
**Non consent care**		
The provider introduces themselves and greeting mother and her support person?	297 (51.70%)	277(48.3%)
The providers encourage mother to ask questions?	368(64.10%)	206(35.9%0
The provider respond mother's question with politeness and truthfulness?	520(90.60%)	54(9.4%)
The providers explain what is being done and what to expect throughout labor and birth?	513(89.40%)	61(10.6%)

Provider gives periodic updates on status and progress of your labor?	505(88.00%)	69(12%)
Providers permit mother to choice of position for birth?	398(69.3%)	176(30.7%)
Health provider can obtain consent or permission prior to any procedure?	458(79.80%)	116(20.2%)
**Non dignified care**		
Health providers shouted at or scolded you?	507(88.30%)	67(11.7%)
Health providers made negative comments about you?	552(96.20%)	22(3.8%)
**Abandonment/neglected care**		
Health providers ignored or abandoned you when you called for help?	516(89.90%)	58(10.1%)
Left unattended during the second stage of labor?	556(96.90%)	18(3.1%)
**Discrimination**		
Health care providers discriminated by race, ethnicity, and economic status?	565(98.40%)	9(1.6%)
Health care providers discriminated because of teenage (< 18 yrs.)?	564(98.30%)	10(1.7%)
Health care providers discriminated because of being HIV positive?	571(99.50%)	3(0.5%)
**Detention in health facility**		
Discharge postponed until hospital bills are paid?	570(99.30%)	4(0.7%)
The woman is never detained or confined Against her will?	569(99.10%)	5(0.9%)
