# Supplementary material for: Prevalence and risk factor for mistreatment in childbirth: In health facilities of Gondar city, Ethiopia
Source: PLoS One. 2022 May 5;17(5):e0268014. doi: 10.1371/journal.pone.0268014 (PMC9070956; doi:10.1371/journal.pone.0268014)
Supplement: S1 Questionnaire — (DOCX) [file pone.0268014.s002.docx]

## Annex III: English Version Questionnaires

| **Part one socio-demographic and economic characters of mother**   \| S no \| Question \| Response \| Skip \| \| --- \| --- \| --- \| --- \| \| 101 \| What is your age? \| ……………. \|  \| \| 102 \| What is your marital status? \| 1. Single  2. Married  3. Divorced  4. Widowed \|  \| \| 103 \| What is your religion? \| 1. Orthodox  2. Catholic  3. Protestant  4. Muslim  5. Other (specify ……. \|  \| \| 104 \| Mother’s level of education \| 1 No read and write  2. Read and write  3. Primary (1-6)  4 Junior (7-8)  5 Secondary (9-12)  6. Collage and above \|  \| \| 105 \| Mother’s occupation \| 1. House wife  2. Private employee  3.Government employee  4. Merchant  5. Student  6. Other (specify)  ____________ \|  \| \| 106 \| Family monthly income (in Ethiopian birr) \| …………….. \|  \| \| 107 \| Place of residence \| 1. Urban  2. Rural \|  \| |
| --- | --- | --- | --- | --- | --- | --- | --- | --- | --- | --- | --- | --- | --- | --- | --- | --- | --- | --- | --- | --- | --- | --- | --- | --- | --- | --- | --- | --- | --- | --- | --- | --- |

**Part 2: obstetrics history of mother**

Now I am going to ask you some questions about your recent delivery in health facility

| 201 | How many live children do you have? | ________ | |  |
| --- | --- | --- | --- | --- |
| 202 | How many times have you ever been pregnant? | ______ | | If answer is 1, (premi) skip to Q 207 |
| 203 | How many times have you ever had abortion (before 7moths of GA)? | ---------------- | |  |
| 204 | How many times have you ever had stillbirth (after 7moths of GA)? | ________ | |  |
| 205 | Did you have ANC visit in health institution for the previous birth? | 1. No 2. Yes | |  |
| 206 | Where did you have previous institutional delivery? | 1. No 2. yes | |  |
| 207 | For how many times did you have ANC follow up for this child? | 0. I had no follow up  1. 1  2. 2  3. 3  4. 4 and above | | If the answer is 0, skip to Q 210 |
| 208 | If you had ANC follow up, where did you have the ANC follow up?  **(Multiple answer is possible)** | 1. Health post 2. Health center 3. Public Hospital 4. Private clinic 5. Other (specify) _______ | |  |
| 209 | \| If you had ANC follow up, who have given you the ANC?  **(Multiple answer is possible)** \| \| --- \| | 1. Doctor  2. Nurse  3. Midwife  4. HEWs  5.Other,specify ___________ | |  |
| 210 | Mode of delivery | 1. Normal delivery  2. Caesarean delivery  3. Vacuum extraction/  forceps delivery  4.Deliveryby episiotomy | |  |
| 211 | For how long you stayed here in the facility after birth? | ___________ (in hour) | |  |
| 212 | Mode of discharge | 1. With professionals recommendation 2. Self (AMA) | |  |
| 213 | Was there any complication during delivery? | 1. No  2. Yes (Mother)  3. Yes (for baby)  4. Yes (both mother and baby) | | If the answer is yes skip to Q 214 |
| 214 | If yes, what was/were the complication/s? | _______________ | |  |
| S no | **1. During your in facility delivery did you experience the following types of physical abuse?** | | 0. No  1. Yes |  |
| 301 | Health provider(s) physically hit, slapped, pushed, pinched or otherwise beat you.? | |  |  |
| 302 | Verbally (insulting) abuse during labor or delivery? | |  |  |
| 303 | Separate mother from baby without  medical indication? | |  |  |
| 304 | Support staffs insult me and my companion? | |  |  |
| 305 | Receiving unnecessary uncomfortable/pain-relief treatment? | |  |  |
| 306 | Denied from food or fluid in labor unless medically necessitated? | |  |  |
|  | **2. During your in facility delivery did you experience this non-confidential care?** | |  |  |
| 307 | The providers didn’t uses drapes or covering appropriate to protect mother’s  Privacy? | |  |  |
| 308 | Health providers discussed your private health information in a way that others could hear? | |  |  |
|  | **3. During your in facility delivery did you experience to get information, informed consent for any procedure** | |  |  |
| 309 | The provider introduces themselves  and Greeting mother and her support  Person? | |  |  |
| 310 | The providers encourage mother to ask questions? | |  |  |
| 311 | The provider responds mother’s question with politeness and truthfulness? | |  |  |
| 312 | The providers explain what is being done and what to expect throughout labor and birth? | |  |  |
| 313 | Provider gives periodic updates on status and progress of your labor? | |  |  |
| 314 | Providers permit mother to choose of position for birth? | |  |  |
| 315 | Health provider can obtain consent or permission prior to any procedure? | |  |  |
|  | **4. During your in-facility delivery did you experience the following types of non-dignified care** | |  |  |
| 316 | Health providers shouted at or scolded you.? | |  |  |
| 317 | Health providers made negative comments about you? | |  |  |
|  | **5. During your in-facility delivery did you experience the following types of abandonment/neglect of care?** | |  |  |
| 318 | Health providers ignored or abandoned you when you called for help? | |  |  |
| 319 | Left unattended during the second stage of labor? | |  |  |
|  | **6. During your in facility delivery did you experience the following types of discrimination** | |  |  |
| 320 | Health care providers discriminated  by race, ethnicity, and economic status? | |  |  |
| 321 | Health care providers discriminated because of teenage (< 18 yrs.)? | |  |  |
| 322 | Health care providers discriminated because of being HIV positive? | |  |  |
|  | **7. During your in-facility delivery did you experience the following types of detention** | |  |  |
| 323 | Discharge postponed until hospital bills are paid? | |  |  |
| 324 | The woman is never detained or confined Against her will? | |  |  |

## Annex VI. Amharic Version Questioner

ክፍልአንድማህበራዊናዱሞግራፊያዊሁኔታዎች

| \| ተ.ቁ \| \| --- \| | ጥያቄዎች | መልስ | እለፊ/ፍ |
| --- | --- | --- | --- | --- |
| 101 | እዴሜዎትስንትነው? | ----------------- |  |
| 102 | በአሁኑሰዓትየጋብቻሁኔታዎምንይመስላል? | 1. ያላገባ  2. ያገቡ  3. የፈታ  4. በሞትየተለየ |  |
| 103 | ሀይማኖትዎምንዴነው? | 1. ኦርቶዶክስ  2. ካቶሊክ  3. ፕሮቲስታን  4. ሙስሊም  5. ልላካለ( ይገለጽ) |  |
| 104 | የትምህርትደረጃዎትንቢገልጽልኝ? | 1.መደበኛየሆነትምህርትያልተማረች  2. ማንበብ እና መፃፍ  3. የመጀመሪያደረጃ (1-4)  4. የመጀመሪያ ደረጃ (5-8)  5. ሁለተኛደረጃ (9-12)  6. ዲፕሎማና ከዛበላይ |  |
| 105 | በአሁንሰአትበምንዓይነትስራነውየተሰማሩት? | 1. የቤት እመቤት  2. የግል ተቀጣሪ  3. የመንግስት ሰራተኛ  4. ነጋድ  5. ተማሪ  6. ልላካለ( ይገለጽ |  |
| 106 | በወርየሚያገኙትገቢምንያህልነው? | በወር-------------- ብር |  |
| 107 | የመኖርያቦታ | 1 ከተማ  2 ገጠር |  |

ክፍሌሁለት፡የእናትየዋየወሊዴታሪክ

| ተ.ቁ | ጥያቄ | መሌስ | እለፊ/ፍ |
| --- | --- | --- | --- |
| 201 | በህይወትያሉስንትልጆችአሉዎት? | ­…………… |  |
| 202 | ምንያህልጊዜእርጉዝነበሩእስካሁን? | …………….. | መልሱ 1 ከሆነ (መጀመሪያ) ወደ Q 207 ይዝለሉ |
| 203 | ምንያህልጊዜውርጃአጋጥሞታል (ከ 7 ወርበፊት)? | ……………. |  |
| 204 | ምን ያህልልጆችከ 7 ወር በሁላ ሞቶቦታል? | ………… |  |
| 205 | ከዚህ በፊት ላለው ልጅዎ የቅድመ ወሊድ( የእርግዝና) ክትትልያደርጉነበር? | 0. የለም  1 አዎ |  |
| 206 | ቀደምሲል ለወዱት ልጅ ወዴት ነው የተወለደው? | 1. ቤት  2. የጤናተቋም  3. ሌላ, ይግለጹ |  |
| 207 | በዚህልጅላይምንያህልጊዜየድህረወሊድ( የእርግዝና) ክትትልያደርጉነበር? | 0 ምንምክትትልአላገኘሁም  1. 1  2. 2  3. 3  4. 4 እናከዚያበላይ | መልሱ 0 ከሆነ, ወደ Q 210 ይለፉ |
| 208 | የቅድመወሊድ( የእርግዝና) ክትትልካደረጉየትነው?  (ብዙመልሶችይቻላል | 1. ጤናከላ  2. ጤናማዕከል  3. የሕዝብሆስፒታል  4. የግልክሊኒክ  5. ሌላ (ይግለጹ |  |
| 209 | የቅድመወሊድክትትል( የእርግዝና) ካደረጉየሰጡትማንነው?  (ብዙመልሶችይቻላል) | 1. ሐኪም  2. ነርስ  3. አዋላጅ  4. የጤናኤክስቴንሽንሰራተኞች  5. ሌላ, ካለያሳውቁ___________ |  |
| 210 | በምንአይነትሁኔታነውየወለዱትየአሁኑንልጅ? | 1. በኖርማል  2. በቀድጥገና  3. በመሳሪያዴጋፍ  4. በስቲች |  |
| 211 | እዚህከወለዱበኋላለምንያህልጊዝቆዩ? | ___________ (በሰዓት) |  |
| 212 | በምንአይነትነውየወጡት ? | 1. በሀኪምትዛዝ  2. በገዛፈቃድ |  |
| 213 | በወልሊድጊዚችግርገጥሞወትነበረ? | 1. አይ  2. አዎ (እናት)  3. አዎ (ለህጻኑ)  4.አዎ(ሁለቱምእናትእናህኑ | መልሱአወከሆነ, ወደጥቁር 214 ይሂዱ |
| 213 | አዎከሆነ, ችግሩ / ምንድንነበር? | ……………………… |  |

ክፍሌሦስት፡አክብሮት የጎደለዉ የወሊዴ አገሌግልት ና ጉዳት በተመለከተ

| ተ.ቁ | 1. **በጤና ተቋም ሲወሌደ አካሊዊ ጉዲትድርሶቦታሌ** | 0. የለም  1. አዎ |
| --- | --- | --- |
| 301 | በምጥናበወሊዴጊዜበጤናባለሙያዎአካላዊጉዳትደርሶብታሌ (ሃይሌመጠቀም፣መደብደብ፣ማጋጨት፣መግፈትር…)? |  |
| 302 | በምጥናበወሊዴጊዜበጤናባለሙያዎበቃላትስድብደርሶብወታል? |  |
| 303 | ከህክምናትእዛዛዉጭከልጄወጋርእንዲለዩተደርገዋል? |  |
| 304 | አንዳንድድጋፍሰጪ(የጽዳት፡የካርድክፍሌ፤የጥበቃ) ሰራተኞችበተለያየምክንያትሰድበወታል? |  |
| 305 | አላስፈላጊወይምየማይመችየህመምማስታገሻህክምናተደርጎለወታል? |  |
| 306 | በህክምናአስፈሊጊሳይሆንከምግብናከመጠጥ  እንዴቆጠቡተደርገዋል? |  |
|  | **2. በጤናተቋምሲወሌደሚስጥራዊነቱንያልጠበቀአገሌግልትአጋጥሞታሌ** |  |
| 307 | ጤናባለሙያውተገቢውንየሆነመከለያልብስተጠቅማል? |  |
| 308 | ጤናባለሙያውየእርስዎንሚስጥራዊመረጃልሎችበሚሰሙትሁኔታሲወያዩነበር? |  |
|  | **3. በጤናተቋምሲወሌዱየእርሶፍቃዯኝነትተጠይቆነበር** |  |
| 309 | ጤናባለሙያውእራሱንአስተዋውቆእናሰላምታሰጥታልለእናትየውእናአብሮአትላለውሰው? |  |
| 310 | ጤናባለሙያውእናትየውጥያቄእንድትጠይቅያበረታታል? |  |
| 311 | ጤናባለሙያውየእናትየውጥያቄበትህትናእውነትላይተመርኩዞይመሌሳል? |  |
| 312 | ጤናባለሙያውበምጥሰዓትምንእየተሰራእንደሆነእናምንእንደሚያጋጥምያብራራል? |  |
| 313 | ጤናባለሙያውበየግዜውየምጡንሂደትያብራራል? |  |
| 314 | ጤናባለሙያውበምጥሰዓትእናትየውእንደተመቻትሆናእንድትወልድይፈቅድነበር? |  |
| 315 | ጤናባለሙያውምንምአይነትአገሌግልትከመሰጠቱበፊትከእናትየውፍቃዴይጠይነበር? |  |
|  | **4. በጤናተቋምሲወልዱእነዚህንክብረነክዴርጊቶችአጋጥሞትነበር** |  |
| 316 | ጤናባለሙያውጮሆቦትወይምገፍትሮትነበር? |  |
| 317 | ጤናባለሙያውእርሶንበሚመለከትመጥፎአስተያትሰጥቶነበር? |  |
|  | **5. በጤናተቋምሲወሌደእነዚህንቸልተኝነቶችአጋጥሞትነበር** |  |
| 318 | ለእርዲታበሚጣሩበትግዜጤናባለሙያውበቸሌተኝነትአሌፎታሌ? |  |
| 319 | ምጥላይእያለልጁበሚወጣበትግዜጤናባለሙያውትተዎትሄዶነበር? |  |
|  | **6. በጤናተቋምሲወሌደእነዚህመድልዎችደርሶውቦትነበር** |  |
| 320 | ጤናባለሙያውበዘር፣ጎሳ፣በሀብትአዴሎአድርገውነበር? |  |
| 321 | ጤናባለሙያውበእዴሜ (18 በተችበመሆንሽ) አድልአዴርገውነበር? |  |
| 322 | ጤናባለሙያውኤች. አይ. ቪስላለብሽብቻአድሎአድርገውነበር? |  |
|  | **7. በጤናተቋምሲወሌደእነዚህቅጣቶችአጋጥሞትነበር** |  |
| 323 | ከሆስፒታሉየሚወጡበትግዜሂሳብበለመክፈልምክንያትዘግይቶነበር? |  |
| 324 | ያለፍቃድምንምአይነትቅጣትአልተደረገቦትም? |  |
