# Supplementary material for: Prevalence and risk factor for mistreatment in childbirth: In health facilities of Gondar city, Ethiopia
Source: PLoS One. 2022 May 5;17(5):e0268014. doi: 10.1371/journal.pone.0268014 (PMC9070956; doi:10.1371/journal.pone.0268014)
Supplement: Attachment — Submitted filename: Response to Reviewers.docx [file pone.0268014.s004.docx]

**To: Editor-in-chief of PLOS ONE Journal**

**Subject: Submission of a revised manuscript for publication**

Dear Editor,

Thank you for allowing us to submit a revised draft of our manuscript entitled *“High attribution of non-consented care for high prevalence of mistreatment among postpartum mothers who give birth at public health facilities in Gondar City, northwest Ethiopia "****[Manuscript ID number: PONE-D-21-04196]****.* We appreciate the time and effort dedicated by you and the reviewers to provide your valuable feedback on our manuscript. We are grateful to the reviewers for their insightful comments which improve our manuscript.

We accepted and tried to incorporate all of the comments provided. Thus, the comments are attached here below with their point-by-point responses given in blue font color. Besides, the detailed changes made are highlighted in the “revised manuscript with track changes” to easily identify the changes/improvements, and also the clean copy of the revised manuscript, questionnaire and STROBE form are prepared. On edition of manuscript all authors, and Tsagye Haile from Belgium, assistant professor of health policy was involved.

**Response to Editor**

1. Please correct the many English typos

Response 1: We authors tried to improve the language including the typos

2. Careful with the interpretation of the reference. In the first paragraph of the introduction, you have reference 1 (The 2014 UNICEF report on maternal mortal between 1990 and 2013). Please find that report to correct your numbers. There is no, in that report, an African MMR. There is a sub-Saharan Africa estimate. And it is 510 per 100,000 live-births; also the fraction of deaths is not 64%, is 61%. [Check that report on table 2 and on page 21].

Response 2: Thank you dear editor, we have checked the reference and evidence documented and found error introduced. Hence, we have correctedit accordingly. Please see a clean version of manuscript page number 4-line number 41-42

3. In the description of the study area please do mention the private clinics as well. Also, do add basic description of coverage of institutional deliveries and antenatal care.

Response 3: Thank you, we have accepted the comments, and considered in the study setting section, page 6 line 91.

4. On sampling procedures please use words to explain what is that k-interval for the systematic sampling. See further comments of the reviewer below.

Response 4: We have accepted all the comments, and corrected all, see clean version of manuscript page 7 line 111.

5. The operational definition section needs a table with the list of things to make these definitions. It would be beneficial if the English instrument were added in the supplements.

Response 5: Thank you, we have accepted, and summarized in table 1, please see under the operational definition, page 8.

6. The data processing and analysis section need to be expanded.

Response 6: Thank you for the comments, we entertained in the data processing and analysis section.

7. We do not analyze a logistic regression. We use logistic regression to analyze something. Please correct.

Response 7: We authors forwarded great thanks; we have addressed it.

8. There is use of confidence intervals for a proportion in the results. How such thing interval was estimated? This is something to put in the methods

Response 8: We have corrected it, see under data process and analysis section.

9. Line 219 is for the “data processing and analysis” section not in the “results”. Please correct.

Response 9: We have corrected it.

10. When report the mean as in the line 163 we usually write the SD as, for example, “(SD = 5.76)”. Notice the equals sign

Response 10: It is corrected based on comment, see page 11 line 162

11. Remove the “The” on “The proportion of mistreatment during facility-based childbirth”

Response 11: We have removed it.

12. Please report the confidence intervals for all mistreatment types in text

Response 12: We authors accepted the comment and we have included confidence interval for all mistreatment types.

13. Comments regarding table 1;
a) Add the mean and SD of the age,
b) Make all percentages 1 decimal place,
c) Add more categories for the “family estimated monthly income” variable. and please provide quartiles.
d) On “HCP conducting ANC follow up” variable please correct the “Privet clinic”. That should be “Private clinic”.
e) Please check the alignment for “complication during delivery”.

Response 13: All the comments are addressed accordingly, see on page 16, table 3.

14. Comments regarding table 3;
a) for the ORs please make all 2 decimal places, make sure that references have 1 (for example it is missing on Educational status)

Response 14 a. All are corrected based on your comments

b) Why age and “religion” are not included? Regardless of the p-value. This is a key variable.

Response 14 b: Thank you for your view, we have included both variables in the revised manuscript, please see in the table 4.

15. Comments regarding figure 1
a) This pie is taking a lot of space to inform exactly the same in text. And it does not provide any confidence interval as in the text you did. Drop it. I suggest to add this information on figure 2.

Response 15: We haveaccepted the suggestion and corrected accordingly

16. Comments regarding figure 2
a) Add the proportion of mistreatment you have on pie. Add this to the bar plot.
b) Add to all of these bar error bars to capture the confidence interval
c) Fix the decimal places to 1
d) Remove the percentage symbol in the plot
e) Remove the so called main title this can be placed in the x-axis and in caption of the plot

Response 16: We have accepted all the comments and correction are made accordingly

17. Discussion
a) Lines 253 to 257. The reason for the differences did you check that these studies define the same way as you do here?

Response 17a: The studies define mistreatment in different ways, for example study conducted in Addis Ababa to define mistreatment were used six categories while in current study used seven categories disrespect and abuse.

b) Are the populations comparable besides the health facility?

Response 17b:

- Beside the study participants, there is variation in proportion of previous history of institutional delivery, in current study 21% of the study participants had no previous history of institutional delivery, in studies conducted Addis Ababa (31%), and Arba Minch (37%). The absence of pervious history of institutional delivery was reported as risk factor for childbirth care mistreatment in current study.
- Thank you, dear editor, for suggestion, we have accepted the suggestion and included under discussion section.

c) Be very careful. We do not want to label medical schools as the center of mistreatment

Response 17c: We apologized for interpretation error. we have accepted the comment and corrected it.

d) What the authors thing of the tool they used to assess the mistreatment? Did you check for validity to use in this setting? Please discuss this. And most likely this must be considered for the limitation subsection.

Response 17d: In this study, mistreatment measurement tool was developed from Maternal and Child Health Integrated Program (MCHIP) respectful maternity care tool kit which was not validated in Ethiopia. We have accepted the comment and acknowledge as limitation in limitation section.

e) The strengths and limitations need more elaboration. This is a health facility-based study therefore a potential for selection bias may doom the generalization of this study. There is always possibility of confounding this must be discussed.

Response 17e: It is corrected based on comments

18. Please fill the STROBE statement and add to the supplements

Response 18: We have filled the STROBE statement and submitted in supplementary file list.

19. Add a specific and clear section of ethics

Response 19: We have includedethics under method section

20. Comments regarding abstract: -
a) OR please make sure they have 2 decimal places
b) For all mistreatment proportion make sure they have confidence intervals

Response 20: We have accepted and have corrected all the comments accordingly

21. Please provide additional details regarding participant consent. In the ethics statement in the Methods and online submission information, please ensure that you have specified what type you obtained (for instance, written or verbal, and if verbal, how it was documented and witnessed). If your study included minors, state whether you obtained consent from parents or guardians. If the need for consent was waived by the ethics committee, please include this information.

Response 21: We have included the comments in ethics statement section. Consent from parents or guardians was not taken because all the study participants have already established their own family that even though few participants are aged below 18 years, they are mature minors. Hence, we did not expected to get consent from their family.

22. In your Methods section, please provide additional information about the participant recruitment method in particular a description of how participants were recruited.

Response 22: We have accepted the description about sampling procedure was shallow in the first submission. In this revised manuscript we have entertained the sampling procedure in detail.

23. You indicated that you had ethical approval for your study. In your Methods section, please ensure you have also stated whether you obtained consent from parents or guardians of the minors included in the study or whether the research ethics committee or IRB specifically waived the need for their consent

Response 23: We have included the comments in ethics statement section. Consent from parents or guardians was not taken because all the study participants have already established their own family that even though few participants are aged below 18 years, they are mature minors. Hence, we did not expect to get consent from their family.

Moreover, according to the Ethiopian national research ethics review guideline, consent from parents or guardians for adolescents aged less than 18 years is waivered for some cases. Among these emancipated/mature minors, one is, those who married or established their own family. Hence, consent was not obtained from parents or guardians for these pregnant adolescents. This information is noted in the main manuscript with citation of the reference.

24. We suggest you thoroughly copyedit your manuscript for language usage, spelling, and grammar.

Response 24: Thank you. We tried to edit the language.

25. During our internal evaluation of the manuscript, we found significant text overlap between your submission and the following previously published works:

- <https://www.biorxiv.org/content/10.1101/430199v1>(Introduction, paragraph 1, sentences 3-5) (Introduction, paragraph 4, sentences 1-2) (Discussion, paragraph 13, sentence 3)

- <https://www.tandfonline.com/doi/full/10.1080/09688080.2018.1502020> (Introduction, paragraph 2, sentence 1)

- <https://www.researchsquare.com/article/rs-107805/v1>(Introduction, paragraph 2, sentence 2)

- <https://preview-reproductive->healthjournal.biomedcentral.com/articles/10.1186/s12978-015-0024-9 (Types of mistreatment during facility-based childbirth, sentences 1-2) (Discussion, paragraph 5, sentence 4)

-https://journals.plos.org/plosone/article?id=10.1371%2Fjournal.pone.0205545(Discussion, paragraph 2, sentence 4) (Discussion, paragraph 12, sentence 4)

- <https://obgyn.onlinelibrary.wiley.com/doi/abs/10.1016/j.ijgo.2014.08.015> (Discussion, paragraph 6, sentence 4)

- <https://bmcpregnancychildbirth.biomedcentral.com/articles/10.1186/s12884-018-1970-3> (Discussion, paragraph 11, sentence 4)

Response 25: We accepted the all comments and we made correction

26. Provide details as to how the current manuscript advances on previous work.

Response 26: We authors believed that this research work adds the following body of knowledge with the existed evidence:

- Evidence from the previous studies focused on hospital-level settings, while the current study involved nine health centers, where disrespect and abuse occur.
- In our study non-confidentiality care is the most experienced form of abuse and respect during health facility childbirth, which is not reported by the previous studies.
- A new factorwas identified by this study such as having complication during childbirth and lack of or less than four antenatal care visits was associated with mistreatment.

**Reviewer 1'**

1. Grammar and spelling: The paper is written in poor English, has multiple grammatical and spelling errors throughout the entire document. I have highlighted most of them in the manuscript. Recommendation: the authors need to rewrite the paper in standard English to enable the readers to understand.

Response 1: Thank you. We tried to edit the language.

2. Justification (line 72-74): the two statements contradict each other. Which is the correct position?

Response 2: Thank you dear reviewer to your comment, we have rewritten the whole paragraph. The lack of information regarding disrespect and abuse in the study area is the correct statement.

3. Study site:
a) it is not clear whether the study was conducted in one or multiple study sites.

Response 2a: Dear reviewer, in this study eight health centers and one compressive specialized hospital were included which means multiple study sites were considered

b) What is the name of the facility?

Response 3b: In this study, all childbirth care providing health facilities; eight health centers, and one referral hospital were considered. Gondar University compressive specialized hospital, Azezo health center, Gondar health center, Merak health center, Woleka health center, Teda health center, Genbot 20 health center, Biajing health center, and Gebreale health center were included in the study. For further information, We haveincluded it under sampling procedure section.

c) How was it chosen from among the other 14 facilities in the city?

Response 3c: In this study, all childbirth care providing health facilities in city; eight health centers, and one referral hospital were considered. The number 14 in study setting section indicate that number of health posts. Health posts currently not provide delivery care (childbirth care).

d) What are the numbers of deliveries handled by this facility per annum?

Response 3d: The average number of deliveries per month in health institutions was 1,173 (Gondar University comprehensive specialized hospital, Azezo health center, Gondar health center, Merak health center, Woleka health center, Teda health center, Genbot 20 health center, Biajing health center, and Gebreale health center had averagely 928, 66, 38, 40, 25, 50, 11,7,8 skilled births per month respectively) (10). The total coverage of institutional deliveries and antenatal care was 62%, and 86% respectively. The number of deliveries of Gondar University comprehensive specialized hospital is high when compared to other facilities, this might be because of referral hospital. We have mention it under study setting and sampling procedure sections.

e) What made this site the most ideal for your study?

Response 3e: the reason to select this site

- The first reason is referral site, Gondar University comprehensive specialized hospital handle high number of deliveries per month (928), high number of client flow might be affecting the quality of care.
- The second reason, in this study site there is high discrepancy between in skill deliveries and antenatal carevisits, it also indicates low quality of care.
- Third reason, in this study area the number of deliveries handledby health centers was low.

4. Sample size: the explanation after sample size calculation is not clear. recommendation: edit for clarity

Response 4: Thank you, we have edited for clarity

5. Comments regarding data collection procedures:
a) Review the whole of this section for consistency in grammar eg the use of "is" instead of "was".

Response 5a: Thank you, we have corrected rigorously

b) Clarify the role of the degree nurse’s vis a vis the principal investigator

Response 5b: The principal investigator oversees all activities of supervisors (degree nurses) and data collectors, whereas supervisors (degree nurses) did supervision of all the data collectors’ activities.

c) What research experience did the six research assistants have?
d) Response 5c: For data collection, we are employed experienced data collectors in data collection and supervision activities from Tseda health science college staffs. These data collectors were priorly participated in different projects in data collection and supervision activities.
e) Were the research assistants employees of the same hospitals or were they independently employed for the study only? how did you address the issue of bias if these nurses worked in the same facilities where they conducted the interviews?

Response 5e: Our study is free from reporting bias because we were independently employed data collectors for data collection from Tseda health science college.

f) Lots of redundancies and repetitions eg questionnaire was pretested (written more than twice)

Response 5f: We authors accepted and have corrected it

6. Comments regarding results:
a) clarify if n=574 or 584(all tables).

Response 6a: Our study, 584 estimated sample size while 574 the number of response (n=574). it was editorial problem; We have corrected it.

b) Table 2: In some of the cells eg previous ANC attendance, the "n" is not 574 as some of the women were carrying a first pregnancy

Response 6b: Thank you, this was typing error from SPSS output in to the table. We have corrected it, please see from table 3.

7. Discussion: some of the explanations are not coherent e.g. why pregnant women who delivered in a hospital before were likely to be mistreated. Review the explanations

Response 7: We authors accepted the comment given and corrected it

8. Strengths and limitations: too brief and not clear

Response 8: We have rewrite and tried to make it clear.

9. Conclusions: Edit for clarity

Response 9: We have made editorial correction

10. Ethics and consent: did the study pose any risks to the study participants? what care was offered to the abused women? were they linked to any care?

Response 10:

- Participating in this study did not pose the study participants to any risk because first, the interview were exit interview; second, the confidentiality and privacy were highly secured.
- At spot of data collection, determining (knowing) abused woman is difficult, because disrespect and abuse measurement is a composite measurement and it need SPSS analysis to identify abused women. However, after analysis and report write up, we present our findings to health facilities health care manager and health sectors to design intervention.

11. Did you obtain parental consent for those below 18 years of age.

Response 11: We didn't obtain consent from respondents' mother because all the respondents establish their own family and gave birth. Hence, though few respondents' age was between 15 years and 18 years old, these women were treated as mature minors that parental consent is not needed. Their consent is enough. Moreover, according to the Ethiopian national research ethics review guideline, consent from parents or guardians for adolescents aged less than 18 years is waivered for some cases. Among these emancipated/mature minors, one is, those who married or established their own family. Hence, consent was not obtained from parents or guardians for these pregnant adolescents. This information is noted in the main manuscript with citation of the reference.

12. Edit the grammar for clarity

Response 12: Thank you. We tried to edit the language.
