# Supplementary material for: Prevalence and risk factor for mistreatment in childbirth: In health facilities of Gondar city, Ethiopia
Source: PLoS One. 2022 May 5;17(5):e0268014. doi: 10.1371/journal.pone.0268014 (PMC9070956; doi:10.1371/journal.pone.0268014)
Supplement: Attachment — Submitted filename: Response to Reviewers.docx [file pone.0268014.s005.docx]

**To: Editor-in-chief of PLOS ONE Journal**

**Subject: Submission of a revised manuscript for publication**

Dear Editor,

Thank you for allowing us to submit a revised draft of our manuscript entitled *“Prevalence and risk factor for mistreatment in childbirth: in Gondar city health facilities, Ethiopia "****[Manuscript ID number: PONE-D-21-04196]****.* We appreciate the time and effort dedicated by you and the reviewers to provide your valuable feedback on our manuscript. We are grateful to the reviewers for their insightful comments which improve our manuscript.

We accepted and tried to incorporate all of the comments provided. Thus, the comments are attached here below with their point-by-point responses given in blue font color. Besides, the detailed changes made are highlighted in the “revised manuscript with track changes” to easily identify the changes/improvements, and clean copy of the revised manuscript are prepared.

**Response to Editor comments**

**Introduction**

1. Line 46 - maternal mortality ratio of 70,000 per 100,000 live births? Line 47 - Why this “please check the number 70,000”.

Response: Thank dear editor for comments, this is typing error, we apologies to error. We have corrected it on revised version of manuscript. The correct statement is “maternal mortality ratio 70 per 100, 0000 live birth”.

2. - Line 58 -” A laboring mother is subjected toa diverse”. Please correct the “toa”

- Line 61 - put a space before “Mistreatment”
- Lines 70 to 74 there are a lot of strange interval mistakes.
- I cannot understand why the percentages are repeated.

Response: we authors accepted all the comments and corrected accordingly.

**Methods**

1. Line 89 - the “KM” should be “Km”

**Response**: Thank for your comment, we have corrected

2. Line 94 - what is this “2011 EFY”? Why not a more recent reference?

**Response**: Thank you editor for clarification question, EFY means Ethiopian finical year, 2011 EFY is equivalent to 2019 G.C., on the revised document we have included Gregorian calendar date.

3. Line 104 - remove this “Finally, the largest sample size was used”

**Response:** Thank for your comment, we have corrected it

4. Line 109 - put space after “.” In the whole document, there is a lot of these.

**Response:** Thank for your comment, we have corrected it

5. One thing I cannot get from this report is where the women were recruited from within the public health facilities. This is an important detail.

**Response:** Thank you editor for clarification question, we were recruited study participants at health before exit. This might be expose to bias, we have tried to acknowledge this bias as limitation in limitation section.

**Results**

- Line 172 - put space after 17.2%.
- Page 17 - when reporting the prevalence, as for example the one in line 201, please write, for example, 171 (29.8%, 95% CI: 25.4 to 32.8%). Revise all proportions on this page including the one in line 197.
- Please report the odds ratio and their confidence intervals with 2 decimal places. This applies to the abstract, page 18 and table 4.
- Line 222 remove the “at P-value of loss than 0.05”. Do not resume associations to a matter of p-values.

Response: we authors accepted all the comments and corrected accordingly, thank you.

**Discussion**

1. Please revise spaces like line 158 after “.”

Response: Thank you dear Editor for your comment, we have corrected the comment.

**Response to Reviewer #2 comments**

1. I would like to suggest a change in title. What do you think about “prevalence and risk factor for mistreatment in childbirth: a survey in a Gondar City, Ethiopia. I think easier to the reader.

Response: Thank you reviewer for comment. We believe that title is an advertising of the work. As a result, we wrote as “high attribution of non-consented care for high prevalence of mistreatment among postpartum mothers who give birth at public health facilities: in Gondar city, northwest Ethiopia” However, to make easy to reader we modify as “Prevalence and risk factor for mistreatment in childbirth: in Gondar city health facilities, Ethiopia”.

**Introduction**

2. Line 43 - Define the initials: Maternal Mortality Ratio (MMR)

Response: Thank you reviewer for comments, we have incorporated the comments under introduction section line 47-49.

3. Line 48 – it’s not 70,000; it is 70.

Line 55 – this - small letter

Response: Thank you dear reviewer for comments, this is typing error, we apologize to error. We have corrected on revised version of manuscript. The correct statement is “maternal mortality ratio 70 per 100, 0000 live birth”.

4. Line 57 and line 72 – disrespect and abuse have different definition from mistreatment. (Bohren et al., 2015; Bowser & Hill, 2010), Line 55 – 61 – define mistreatment in childbirth or disrespect and abuse. (Bohren et al., 2015; Bowser & Hill, 2010)

Response: Thank you reviewer for constructive comments, we define mistreatment under introduction section line 39-41.

5. Line 69 - three out of four/ three in four

Line 70 - four hundred twenty mothers out of / in one hundred thousand live 71 birth

Response: Thank you for suggestion, we have accepted comments and corrected.

6. The first paragraph - I think the first paragraph is important to explain Africa and Ethiopia's context. However, this paragraph is not about the major issue (mistreatment). I suggest starting the manuscripts by talking about mistreatment and put information about maternal mortality throughout the introduction.

Response: Thank you reviewer for your valuable comments, we have tried to incorporate all comments, please see first paragraph under introduction section.

7. The introduction talking about mistreatment as a strategy to prevent maternal mortality. However, mistreatment should cause death in a small part of victims. It’s mean that to prevent maternal mortality other issues should be addressed before as antenatal care and health assistance in labor/childbirth, for example. Mitigate mistreatment is important to improve women's human rights and decrease the risk of consequences as postpartum depression and evasion of health services (Leite et al., 2021; Leite, Pereira, Leal, & Silva, 2020; Silveira; et al., 2019). Another possibility is explaining better that women have afraid to go to the hospital due to mistreatment and this behavior puts women at risk to die.

Response: Thank you reviewer for your valuable comments, we have made modification, see line 74-75

8. What are the risk factors to suffer mistreatment in Ethiopia or Africa? Nothing about this was informed.

Response: Thank you for your comments, we have summarized risk factors to mistreatment in Africa under line 86-88.

**Methods section**

1. In “population” is not clear the hospital (s) participants. The research was conducted in one hospital because there is only one hospital in the city? Or there were other Hospitals/health services? I think this situation could be clearest in the text. Manuscript: In “Population” – “All postpartum mothers who gave birth at public health facilities of Gondar city during a period 93 of data collection were considered as the study population.”

Response: Thank you dear reviewer for your clarification question, in Gondar city nine health facilities (one hospital and eight health centers) provide delivery service, in our study all delivery service providing health facilities were included.

2. Manuscript: In “Sample size determination and Sampling Procedure” – “Finally, to get the study 100 participants systematic sampling method with k-interval of two were employed at each facility” I cannot understand the sampling. It was the census of all women in postpartum between March and April 2019 or it was a sampling of all eligible women.

Response: Thank you again for your clarification question. To get study participants from each delivery service providing health facilities. First, the estimated sample size was proportionally allocated to health facilities (the eight health centers and one referral hospital) based on their average monthly deliveries number. Then, finally, to get study participants (allocated sample size) from each facility a systematic sampling technique with two k intervals employed, until required sample size attains.

3. Line 102 – 127 - The authors could to presents the original question which was asked for women

Response: Thank you dear reviewer for your comments, during first review the editor and first reviewer request us to supplement the questionnaire, as per request we had already submitted the questionnaire as supportive file.

4. The topic “Operational definition” should be before “Data Collection Tool and procedures” That is, a total 136 of 24 verification criteria of mistreatment were included.” Should be in topic “Operational definition”

Response: Thank for comments, we have corrected the comments accordingly.

5. The line the topic “Operational definition” should describe all variables included in the analysis.

Response: Thank you dear reviewer for your comment, we have described all variables under operational definition section, see line 130-140

6. There no information about ethics committee approval

Response: Thank you reviewer for your suggestion, we have already state about ethics under methods section, page 11 line 175-187

**Results section**

1. Table 1 – income: inform the currency

Line 218 – 220 – I suggest change this sentence to “statistical Analysis”

Response: Thank you reviewer for your comments, we have corrected it.

2. Comments: Commonly, the choices about variables that compose the multivariate logistic regression have been done based on statistical criteria. But, in my point of view, this choice should be based on in theoretical model using the DAG definition about causality and confounders.

Response: Thank you for comments, we added variables in multivariate logistic regression, based on their statistical criteria, in additional to this, significantly associated variables in previous studies were included in multivariate logistic regression analysis without considering statistical criteria.

3. Based on my own country, type of birth and parity are important predictors of mistreatment in labor. I suggest adding this information to the model.

Response: Thank dear reviewer for your suggestion, we have added both variables (type of birth and parity) in the model irrespective of their p-value.

4. history of previous institutional delivery is a complicated variable. This should be used only if a woman is multiparous. To primiparous women, this question is not applicable. Therefore, this variable just could be used in a sensitivity analysis with multiparous women.

Response: Thank you for your comments, we have removed complicated variable (history of previous institutional delivery) from model to avoid effect and we did reanalysis.

5. Suggests: I suggest conducted some multivariate logistic regression stratifying by parity and type of birth.

Response: Thank you for your valuable comments, we have done some logistic regression stratification analysis by parity and type of birth, please see, line 251-261, table 5 &6 in revised version of manuscript.

**Discussion:**

1. In my opinion, the discussion about prevalence should offer more than a simple comparison between the current study and the other studies. The context of the hospitals should be considering and why health care providers use to mistreat women in Ethiopia. The prevalence was very high.

Response: thank you for your comments, as I early mentioned, our study is more focused on evaluating health care provider and women behavior related mistreatment dimensions, hospital and health facility environment constrain were not assessed, due to this reason we can’t support our finding with health system and facility environment related reasons. We accepted it as limitation

2. Considering the discussion about risk factors, the authors could explain why (the causal mechanism) some variables present as a risk factor. Comparison with other studies is important to observe some consistency of the findings. However, the interpretation of the finding should be the major topic.

Response: Thank you for comments, we have tried to explain causal mechanism for some risk factors, please see discussion section.

Strength and limitation

1. I have some doubts if collect information immediately following delivery and inside the hospital/health center is a good option to measure mistreatment. Some women could have been afraid to address this issue and suffer consequences (more mistreatment) due to these acts. A second point is some women could present the “gratitude bias” to be alive and with their baby in arms. Thereby, only a few days after the delivery they get realize all violence and mistreatment that she has suffered. Do not address fundal pressure and episiotomy could be a limitation.

Response: Thank your dear reviewer for your constrictive comments, we have accepted all the comments and we have tried to acknowledged all these limitation under limitation section.

Conclusion

1. Knowing the mistreatment prevalence is high, what action it is possible to take aiming to mitigate mistreatment in Ethiopia?

Response: Thank you reviewer for comments, to alleviate mistreatment health mangers need to strengthening actions, like providing maternity education during antenatal care and appropriate management of complications to improve the quality of maternity care at health facilities. Also, health care providers that work in childbirth care need to be trained on the importance of informed consent and give compassionate and respectful care. Moreover, Health facilities need to promote positive birth experiences through the provision of respectful, dignified, supportive, and consented care. We state this recommendation under conclusion section.

**References:**

Bohren, M. A., Vogel, J. P., Hunter, E. C., Lutsiv, O., Makh, S. K., Souza, J. P., … Gülmezoglu, A. M. (2015). The Mistreatment of Women during Childbirth in Health Facilities Globally: A Mixed-Methods Systematic Review. PLoS Medicine, 12(6), 1–32. https://doi.org/10.1371/journal.pmed.1001847

Bowser, D., & Hill, K. (2010). Exploring Evidence for Disrespect and Abuse in Facility-Based Childbirth Report of a Landscape Analysis. Harvard School of Public Health University Research Co., LLC. https://doi.org/10.1624/105812410X514413

Leite, T. H., Gomes, T., Marques, E. S., Pereira, A. P. E., Silva, A. A. M. da, Nakamura-Pereira, M., & Do Carmo Leal, M. (2021). Association Between Mistreatment of Women during Childbirth and Postnatal Maternal and Child Health Care: Findings from “Birth in Brazil.” Women and Birth.

Leite, T. H., Pereira, A. P. E., Leal, M. do C., & Silva, A. A. M. da. (2020). Disrespect and abuse towards women during childbirth and postpartum depression: findings from Birth in Brazil Study. Journal of Affective Disorders, 273, 391–401.

Silveira;, M. F., Mesenburg;, M. A., Bertoldi;, A. D., Mola;, C. L. De, Bassani;, D. G., Domingueses;, M. R., … Coll, C. V. N. (2019). The association between disrespect and abuse of women during childbirth and postpartum depression: Findings from the 2015 Pelotas birth cohort study. Journal of Affective Disorders, 256(April), 441–447. <https://doi.org/10.1016/j.jad.2019.06.016>

Response: Thank you dear reviewer for your references suggestion, the references are too helpful to improve our manuscript, we used all references.
