# Supplementary material for: Prevalence and risk factor for mistreatment in childbirth: In health facilities of Gondar city, Ethiopia
Source: PLoS One. 2022 May 5;17(5):e0268014. doi: 10.1371/journal.pone.0268014 (PMC9070956; doi:10.1371/journal.pone.0268014)
Supplement: Attachment — Submitted filename: Response to Reviewers.docx [file pone.0268014.s006.docx]

To: **Editor-in-chief of PLOS ONE Journal**

**Subject: Submission of a revised manuscript for publication**

Dear Editor,

Thank you for allowing us to submit a revised draft of our manuscript entitled “Prevalence and risk factor for mistreatment in childbirth: in health facilities of Gondar city, Ethiopia "[Manuscript ID number: PONE-D-21-04196R2]. We appreciate the time and effort dedicated by you and the reviewers to provide your valuable feedback on our manuscript. We are grateful to the reviewer for their insightful comments which improve our manuscript.

We accepted and tried to incorporate all of the comments provided. Thus, the comments are

attached here below with their point-by-point responses given in blue font color. Besides, the

detailed changes made are highlighted in the “revised manuscript with track changes” to easily identify the changes/improvements, and clean copy of the revised manuscript are prepared.

**Response to Reviewer #3 comments**

1.) Suggest editing title to “Prevalence and risk factors for mistreatment in childbirth: in health facilities in Gondar City, Health facilities, Ethiopia

Response: Thank you reviewer for comment. To make easy to reader we modified the title as “Prevalence and risk factor for mistreatment in childbirth: health facilities of Gondar city, Ethiopia”

2.) Abstract-Background section:

a. Is compassionate RMC ensuring women’s survival? I’m unsure of this statement although I do understand that more positive experiences of care could lead to better health outcomes for women and newborns (based on improving the coverage of facility deliveries).

b. You say that compassionate and respectful maternity care has received less attention both in practice and research in Ethiopia I think there is quite a bit of literature about RMC in Ethiopia. Perhaps there is less data from Gondar City specifically and if so, I might state that.

Response for a&b: Thank you reviewer for comments, we have accepted the both comments and we made correction accordingly, please see clean version of manuscript line 15-16.

3.) Abstract—Methods

a. How many facilities was this study conducted in?

Response: Thank you for your clarification question. In this study 9 health facilities are considered. We have stated this in the abstract section, see line 20.

b. These are postpartum women—how long postpartum?

Response: Thank you again reviewer for your clarification question. In this study the postpartum period considered was giving birth to 24 hours.

c. You say: A binary logistic regression analysis was done to see the association between mistreatment and the independent variables. Suggestion: A binary logistic regression analysis was done to see whether there was an association between mistreatment and independent variables such as XXXXXX

d. You say: Finally, the logistic regression analysis was done by stratifying type of party and mode of delivery. I think you mean parity?

Response for c&d: Thank you reviewer for suggestion. We have accepted all the comments and rewrite the paragraph.

4.) Abstract—Results

a. Suggest changing the word “Result” to “Results” on line 27

b. Line 31—change “complication” to “complications” on line 31

Response a&b: Thank for your comments, we have corrected the comments. Please see line 24 and 29.

c. Did you adjust for facility-based clustering?

Response: Thank your reviewer for clarification question, we did not adjusted for facility based clustering because in this study, all childbirth care providing health facilities; eight health centers, and one referral hospital were considered and we didn’t not use cluster sampling method

5.) Abstract—Conclusions

a. Suggest capitalizing the word “the” in line 34

b. The conclusion that the proportion of women who were mistreated was higher here than in other developing countries—it seems like it would be useful to compare what you found with other studies in Ethiopia since there have been quite a few published

Response for a&b: Thank you reviewer for your valuable comments, we have made modification, see line 30-31

c. You mention some possible ways to address mistreatment based on your findings. How would you address the issue that mistreatment was more prevalent in hospitals?

Response: Thank you dear reviewer for your question. To address these issues enhancing health workers capacity through trainings like compassionate and respectful maternity care might solves the problem. Please see conclusion section, we have accepted and add the comment in conclusion section.

d. In your conclusion you state that the proportion of mistreatment was higher in this study but the in the introduction of the paper, you do show ranges of up to 98.9%

Response: Thank you reviewer for your valuable comments, we have made modification, see line 30-31

Introduction

1.Line 43: Maternal mistreatment at childbirth is more than just what you’ve mentioned here. I suggest using a more accepted definition of RMC

Response: Thank you reviewer for your valuable comments, we have made modification, see line 39-42

Methods

2. Line 152: Data were collected through face-to-face interviews. Were these interviews conducted in the health facilities?

Response: Thank you reviewer for your clarification question. The interview was conducted in the health facilities after giving birth before exit.

2. A chart review was also done but which data/indicators were extracted from the charts?

Response: Thank you for your clarification question. Record reviews were done to extract obstetric related variables such as parity, gravidity, number of antenatal care visits, mode of delivery. We have now incorporated these comments, please see line 153.

Results

1. Line 203—The majority of participants had ANC visit—I assume this is at least one ANC visit?

Response: Thank you reviewer for your valuable comments, we have made correction accordingly, see line 205

2.Line 245—You state that mothers who had less than 4 ANC visits were 3.58 times more likely to mistreat—but should be changed to experience mistreatment rather than mistreat; this is the same for the next few sentences—the women themselves are not mistreating but are rather being mistreated

3.Line 251—"The analysis was stratified by party…”; this should be parity

Response for 2&3: Thank you reviewer for your suggestion, we have made correction accordingly, see line 247-252

Conclusions:

1.Line 277—You say here that the findings in this paper show a lower proportion of mistreatment than other studies in Ethiopia so this should be mentioned in the abstract rather than what you’ve mentioned about it being higher

Response: Thank you reviewer for your valuable comments, we have made modification, see line 30-31
